# Active Prior Tactile Knowledge Transfer for Learning Tactual Properties of New Objects

**DOI:** 10.3390/s18020634

**Published:** 2018-02-21

**Authors:** Di Feng, Mohsen Kaboli, Gordon Cheng

**Affiliations:** Institute for Cognitive Systems (ICS), Technische Universität München, Arcisstrasse 21, 80333 München, Germany; fengdi1015@gmail.com (D.F.); gordon@tum.de (G.C.)

**Keywords:** tactile sensing, artificial robotic skin, active tactile object perception, active tactile object learning, active tactile transfer learning

## Abstract

Reusing the tactile knowledge of some previously-explored objects (prior objects) helps us to easily recognize the tactual properties of new objects. In this paper, we enable a robotic arm equipped with multi-modal artificial skin, like humans, to actively transfer the prior tactile exploratory action experiences when it learns the detailed physical properties of new objects. These experiences, or prior tactile knowledge, are built by the feature observations that the robot perceives from multiple sensory modalities, when it applies the pressing, sliding, and static contact movements on objects with different action parameters. We call our method Active Prior Tactile Knowledge Transfer (APTKT), and systematically evaluated its performance by several experiments. Results show that the robot improved the discrimination accuracy by around 10% when it used only one training sample with the feature observations of prior objects. By further incorporating the predictions from the observation models of prior objects as auxiliary features, our method improved the discrimination accuracy by over 20%. The results also show that the proposed method is robust against transferring irrelevant prior tactile knowledge (negative knowledge transfer).

## 1. Introduction

### 1.1. Motivation

We humans perceive tactual properties of an object (e.g., stiffness, texture, temperature, weight) by applying exploratory actions (e.g., pressing, sliding, static contact, lifting) [1]. After applying different exploratory actions on an object, we can obtain its different tactile information. Conversely, making the same exploratory action on different objects produces different tactile observations. Therefore, when we learn about an object, we always link its physical properties with the exploratory actions that we apply on it.

Besides different kinds of exploratory actions, the tactile information we perceive from an object is also dependent on how we apply an action. Consider an example of pressing on two objects. Object 1 is made of soft sponge, and object 2 is made by covering a solid metal with a soft sponge surface. When pressing our fingertips on both objects with a small normal force, we can recognize similar object deformations. However, if we press with a larger normal force, object 1 deforms much more than object 2, since we have reached the metal part of object 2. A similar situation occurs when we apply the sliding movement on object surfaces with different forces and velocities. As a result, by applying different exploratory actions in different ways, we can build a detailed knowledge of the object’s tactual properties which we call tactile exploratory action experiences.

We humans learn about new objects in an active and incremental way. We actively select the most informative exploratory actions to interact with them [2,3]. More importantly, we relate these new objects with the experiences of exploring objects that we have previously encountered. By transferring the prior tactile knowledge, or prior tactile exploratory action experiences, we can largely reduce the amount of exploratory actions required to discriminate among new objects. In this way, we humans save a lot of time and energy, and recognize new objects with high accuracy [4,5,6,7,8,9,10].

Can robotic systems with a sense of touch also perform like humans to actively transfer the past tactile exploratory action experiences when learning about new objects (transfer learning)?

### 1.2. Background

Over the past decades, researchers have developed various tactile sensors and mounted them on robotic systems (e.g., [11,12,13,14,15,16,17]). In this way, the robots with a sense of touch can perceive different objects’ tactual properties by applying exploratory actions. For example, a robot can slide its sensory parts on objects to sense their textural properties [18,19,20,21], establish a static contact to estimate the temperature [22], or lift objects to measure their center of mass [23]. Bhattacharjee et al. [24] developed algorithms to classify objects into four categories: (1) Hard-Unmoved; (2) Hard-Moved; (3) Soft-Unmoved; and (4) Soft-Moved using One Nearest Neighbor Classifier, Hidden Markov Models and Long Short Term Memory networks based on features of time-varying tactile sensor data (maximum force, contact area, and contact motion). Furthermore, several methods have been proposed for the active object exploration problem, in which the robot actively applies multiple exploratory actions to recognize objects (e.g., [25,26,27,28,29,30,31,32]).

However, the problem of transferring the robotic tactile knowledge has been rarely investigated. Even though many transfer learning techniques have been successfully applied to several areas (e.g., Natural Language Processing: [33]; WiFi-based localization: [34]; Computer Vision: [35,36,37,38]; Bio-informatics: [39]), it is our works that introduced tactile transfer learning. Previously, Kaboli et al. [20,21] developed a novel textural descriptor. Using the descriptor, a ShadowHand dexterous robotic hand equipped with BioTac sensors on its fingertips could efficiently discriminate among object surface textures. Later, we designed a transfer learning method [40,41,42] so that the robotic hand could reuse the prior texture models from 12 objects to learn about the surface textures of 10 new objects. However, since only the sliding movement was applied, the robot could only transfer the object textural properties.

In our previous works [43,44], we proposed an active touch learning method in which an UR10 robotic arm with an artificial skin on its end-effector or fingertips could apply sliding, pressing, and static contact movements to learn about objects’ surface texture, stiffness, and thermal conductivity, respectively. Even though our active learning method enables the robot to efficiently learn about objects, the robot still needs to learn from scratch given a new set of objects. In this regard, recently, for the first time in robotics and tactile domains, we proposed an algorithm called Active Tactile Transfer Learning (ATTL) [45] to actively transfer multiple physical properties of prior objects. Using ATTL, the UR10 robotic arm could actively select prior knowledge to transfer (surface texture, stiffness, and thermal conductivity by applying sliding, pressing, and static contact movements). As a result, the robot could use fewer training samples (even one sample) to achieve higher recognition rate, when it learns about new objects.

The robotic systems in the above-mentioned works only applied exploratory actions with fixed action parameters, e.g., sliding with a fixed velocity to perceive surface textures. In order to learn their detailed physical properties (e.g., the vibro-tactile feedbacks by sliding at different speeds) so as to better discriminate among them, the robots should be able to apply exploratory actions with different action parameters.

### 1.3. Contribution

**Figure 1:**
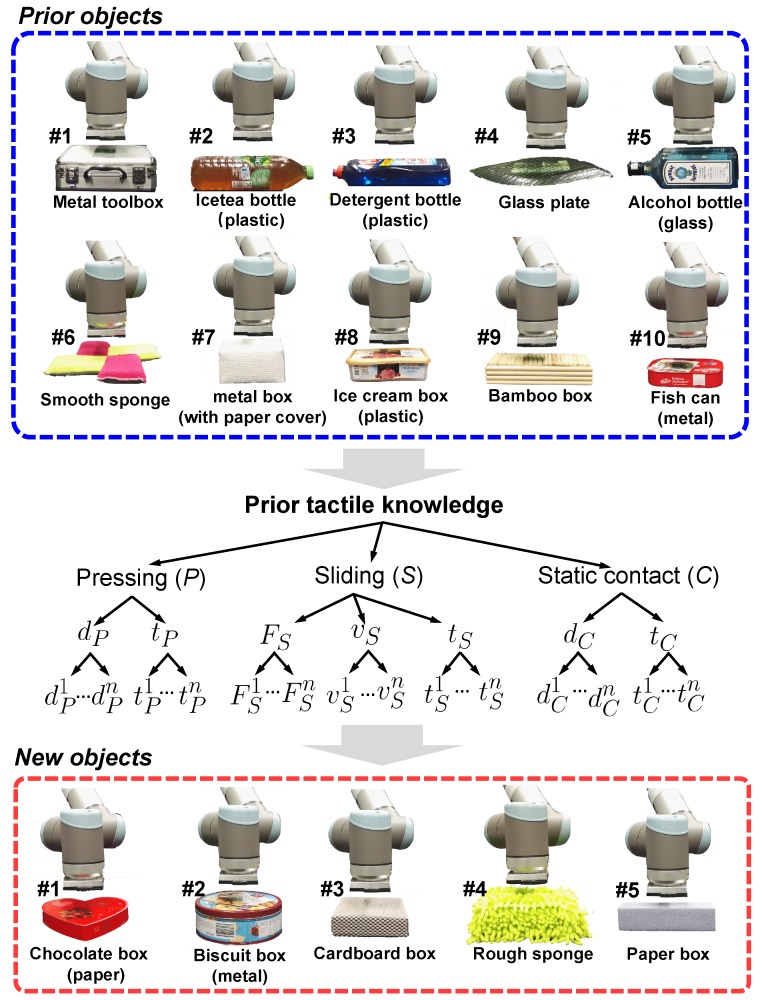
The robot leverages the prior tactile exploratory action experiences built by applying the pressing, sliding, and static contact movements with different action parameters on the prior objects (with index #1–#10) to learn about new objects’ (with index #1–#5) physical properties. The feature observations of prior objects (prior tactile instance knowledge) were used to transfer the action experiences.

In this paper, we focus on actively transferring the prior tactile exploratory action experiences to learn more details about the physical properties of new objects (see Figure 1). Our contributions are two-fold:

- We enable a robot to apply exploratory actions with multiple action parameters. In this way, the robot gains more detailed tactile information.
- We propose an active tactile transfer learning algorithm so that the robot leverages the previously obtained detailed tactile knowledge (prior tactile exploratory action experiences) while learning about a new set of objects.

In the sequel, we first introduce the robotic system (Section 2). Then, we illustrate how the robot applies exploratory actions and obtains the physical properties of objects (Section 3). Afterwards, we illustrate our proposed tactile transfer learning in detail (Section 4), followed by a systematic evaluation of the method (Section 5). We finalize this paper with a conclusion and a discussion about future works (Section 6).

## 2. System Description

### 2.1. Multi-modal Artificial Skin

**Figure 2:**
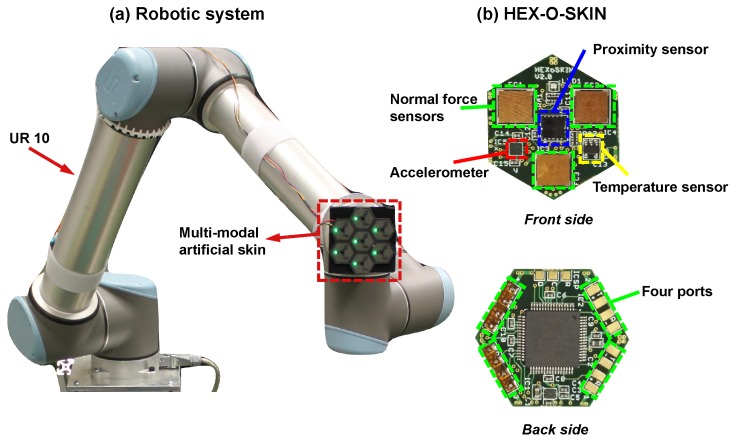
(**a**) The robotic arm equipped with a multi-modal artificial skin; (**b**) The multi modal artificial skin.

To enable the robot to perform more human-like behaviours with multiple tactile sensing modalities, we designed and manufactured multi-modal artificial skin (Figure 2a made by seven active tactile modules (Figure 2b [12]. Each module is a small hexagonal printed circuit board equipped with off-the-shelf sensors (one temperature sensor, one accelerometer, three normal force sensors, and one proximity sensor). In this way, robots are equipped with such an artificial skin that contains seven temperature sensors, seven accelerometers, 21 normal force sensors, and seven proximity sensors. They can emulate the human tactile sensing about temperature, vibrations, force, and light touch. Their technical information is summarized in Table 1.

### 2.2. UR10 Robotic Arm

We mounted the multi-modal artificial skin on the end-effector of an Universal Robotic Arm (UR10) with six DoFs (Figure 2a). The UR10 was controlled in collaboration with the aritificial skin in order to apply different exploratory actions on objects.

## 3. Exploratory Actions and Perception

### 3.1. Exploratory Actions Definition

By applying exploratory actions on objects with different action parameters, the robot can attain different feature observations. In this work, we consider three types of exploratory actions: *pressing* (denoted as *P*), *sliding* (denoted as *S*), and *static contact* (denoted as *C*). Formally, we define $N_{\alpha }$ number of exploratory actions as $A={\left\{\alpha_{n}^{\theta_{n}}\right\}}_{n=1}^{N_{\alpha }}$, where $\theta_{n}$ is the action parameters that define "how" the robot can apply the exploratory action. We further define $\theta_{n}\in \left\{\theta_{P},\theta_{S},\theta_{C}\right\}$, where $\theta_{P},\theta_{S}$, and $\theta_{C}$ represent the action parameters for the pressing, sliding, and static contact movements respectively.

#### 3.1.1. Pressing

**Figure 3:**
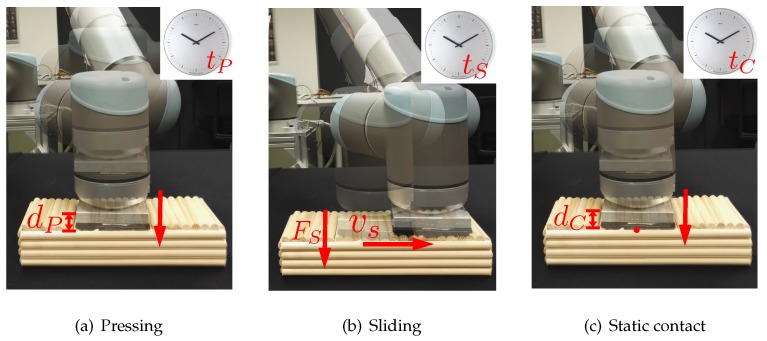
The figure visualizes multiple exploratory actions. (**a**) The pressing movement defined by the action parameters $d_{P}$ and $t_{P}$; (**b**) The sliding movement with action parameters $v_{S}$, $F_{S}$, and $t_{S}$; (**c**) The static contact movement defined by $d_{C}$ and $t_{C}$.

The robotic system presses its sensory part on the object surfaces in order to perceive its stiffness (see Figure 3a). The pressing movement consists of pressing until a depth of $d_{P}$ and holding the artificial skin for $t_{P}$ seconds, i.e., $\theta_{P}=\left[d_{P},t_{P}\right]$. During the pressing, the multi-modal artificial skin can record the normal force feedbacks from each normal force sensor: $F_{n_{f},n_{s}}={\left\{F_{n_{f},n_{s}}^{m}\right\}}_{m=1}^{t_{P}\cdot f_{s}}$ in order to measure the object stiffness. $n_{f}$ is the index of a normal force sensor in one skincell ($n_{f}=1,\ldots ,N_{f}$, in our case $N_{f}=3$), and $n_{s}$ is the index of skincells in the artificial skin ($n_{s}=1,\ldots ,N_{s}$, in our case $N_{s}=7$). $f_{s}$ is the sampling rate of the artificial skin, and *m* the sampling time step. In addition to the normal force feedbacks, the robot can also record the temperature feedbacks from each temperature sensor in order to measure the object thermal conductivity: $T_{n_{t},n_{s}}={\left\{T_{n_{t},n_{s}}^{m}\right\}}_{m=1}^{t_{P}\cdot f_{s}},n_{t}=1,\ldots ,N_{t}$, with $N_{t}$ being the number of temperature sensors in one skincell (in our case $N_{t}=1$).

#### 3.1.2. Sliding

The robot slides the artificial skin on the object surface and perceives its textural properties [18,21] (see Figure 3b). To do this, the robot first builds a contact with objects with the normal force of $F_{S}$, then it linearly slides on the objects with a speed of $v_{S}$ for $t_{S}$ seconds, $\theta_{S}=\left[F_{S},v_{S},t_{S}\right]$. During sliding, the robot collects the outputs of accelerometers (in three axes: x,y,z): $a_{n_{a},n_{s}}^{\left(x\right)}={\left\{a_{n_{a},n_{s}}^{(x),m}\right\}}_{m=1}^{t_{S}\cdot f_{s}}$, $a_{n_{a},n_{s}}^{\left(y\right)}={\left\{a_{n_{a},n_{s}}^{(y),m}\right\}}_{m=1}^{t_{S}\cdot f_{s}}$, $a_{n_{a},n_{s}}^{\left(z\right)}={\left\{a_{n_{a},n_{s}}^{(z),m}\right\}}_{m=1}^{t_{S}\cdot f_{s}}$. Then the robot combines these signals together: $a={\left\{a_{n_{a},n_{s}}\right\}}_{n_{a}=1,n_{s}=1}^{N_{a},N_{s}}$; $a_{n_{a},n_{s}}=\left[a_{n_{a},n_{s}}^{\left(x\right)},a_{n_{a},n_{s}}^{\left(y\right)},a_{n_{a},n_{s}}^{\left(z\right)}\right],n_{a}=1,\ldots ,N_{a}$, where $N_{a}$ is the number of accelerometers in one skincell (in our case $N_{a}=1$). Besides, the change of temperature during sliding is also collected as an extra information $T_{n_{t},n_{s}}={\left\{T_{n_{t},n_{s}}^{m}\right\}}_{m=1}^{t_{S}\cdot f_{s}}$.

#### 3.1.3. Static Contact

The object thermal cues can be attained by the robotic system by applying static contact movement: the robot presses its sensory part against the object surface until a depth of $d_{C}$ and maintains the contact for $t_{C}$ seconds, i.e., $\theta_{C}=\left[d_{C},t_{C}\right]$ (see Figure 3c). The normal force feedbacks and temperature feedbacks are recorded: $F_{n_{f},n_{s}}={\left\{F_{n_{f},n_{s}}^{m}\right\}}_{m=1}^{t_{C}\cdot f_{s}}$, $T_{n_{t},n_{s}}={\left\{T_{n_{t},n_{s}}^{m}\right\}}_{m=1}^{t_{C}\cdot f_{s}}$.

### 3.2. Object Physical Properties Perception

#### 3.2.1. Stiffness

We use the normal force averaged over all normal force sensors and time steps as an indicator for the object stiffness. For the pressing movement with pressing time steps $t_{P}\cdot f_{s}$, object stiffness can be estimated by $\bar{F}=\frac{1}{t_{P}\cdot f_{s}}\frac{1}{N_{f}}\frac{1}{N_{s}}\sum_{m=1}^{t_{P}\cdot f_{s}}\sum_{n_{f}=1}^{N_{f}}\sum_{n_{s}=1}^{N_{s}}F_{n_{f},n_{s}}^{m}$.

#### 3.2.2. Textural Property

In this work, we use the same textural feature extraction method in [43]: The vibration signals a in the artificial skin are used to calculate the activity, mobility and complexity features, denoted as A(a), M(a), C(a). These features represent the object tactile properties in the time domains. We also computed the linear correlation of accelerometer signals between different directions (xy,yz,xz) denoted as L(a), as these accelerometer components are correlated with each other during the sliding movement. The final descriptor of textural features combines activity, mobility, complexity and linear correlation together [43]: TD=[A(a),M(a),C(a),L(a)].

#### 3.2.3. Thermal Conductivity

To extract the features that describe the object thermal cues, we first calculate the average temperature sequence from all the temperature sensors: $\bar{T}=\sum_{n_{t}=1}^{N_{t}}\sum_{n_{s}=1}^{N_{s}}\frac{T_{n_{t},n_{s}}}{N_{t}\cdot N_{s}}$. We then calculate its gradient at each time step as: $\nabla \bar{T}$, and combine it with the average temperature sequence: $\left[\bar{T},\nabla \bar{T}\right]$. To avoid the curse of dimensionality, we further reduce this combination to 10 dimensions via Principle Component Analysis (PCA) method and use it as the final feature to describe the object thermal conductivity.

Table 2 summarizes the exploratory actions, the sensory feedbacks and the corresponding tactile features.

## 4. Transferring Prior Tactile Exploratory Action Experiences

This section describes our proposed active prior tactile knowledge transfer algorithm (APTKT) in detail. First, we formulate our problem in Section 4.1. Then, we illustrate our transfer learning method, including its process (Section 4.3) and the problems of what to transfer (Section 4.4), how to transfer (Section 4.5), from where to transfer, and how much to transfer (Section 4.6). The motivation of our method is demonstrated in Figure 1.

### 4.1. Problem Formulation

Assume that a robotic system has gained prior tactile knowledge of some *old* objects, on which the robot has previously applied different exploratory actions with different action parameters. These prior exploratory action experiences consist of the feature observations perceived by the multiple sensors and observation models from the old objects. Now, the robot is tasked to learn about a set of *new* objects. Since the old objects might share some similar physical properties with the new objects, by leveraging the related tactile exploratory action experiences, the robot can learn about new objects more efficiently.

We define $N_{\mathrm{new}}$ number of new objects ($C^{\mathrm{new}}={\left\{c_{j}^{\mathrm{new}}\right\}}_{j=1}^{N_{\mathrm{new}}}$) the robot is tasked to learn about through different exploratory actions $A={\left\{\alpha_{n}^{\theta_{n}}\right\}}_{n=1}^{N_{\alpha }}$ (For simplicity, we will denote α as an exploratory action in the rest of the paper). In other words, the robot should actively attain object feature observations ($V_{\alpha }^{\mathrm{new}}=\left\{V_{c_{1}}^{\mathrm{new}},V_{c_{2}}^{\mathrm{new}},\ldots ,V_{c_{N_{\mathrm{new}}}}^{\mathrm{new}}\right\}$) for each exploratory action α and construct reliable observation models $V_{\alpha }^{\mathrm{new}}\overset{f_{\alpha }^{\mathrm{new}}}{\to }C^{\mathrm{new}}$. We further define the robot prior tactile experience for an exploratory action α for $N_{\mathrm{old}}$ number of prior objects ($C^{\mathrm{old}}={\left\{c_{i}^{\mathrm{old}}\right\}}_{i=1}^{N_{\mathrm{old}}}$) as the prior object feature observations ($V_{\alpha }^{\mathrm{old}}=\left\{v_{c_{1}}^{\mathrm{old}},V_{c_{2}}^{\mathrm{old}},\ldots ,V_{c_{N_{\mathrm{old}}}}^{\mathrm{old}}\right\}$) and the observation models of old objects $V_{\alpha }^{\mathrm{old}}\overset{f_{\alpha }^{\mathrm{old}}}{\to }C^{\mathrm{old}}$. These feature observations are collected by the multiple tactile sensors from the artificial robotic skin.

We formulate our problem as the transfer learning in the Gaussian Process Classification (GPC) framework [46], where each object is regarded as a class, and for each exploratory action, a GPC model is built as the observation model. The robot iteratively applies the exploratory actions and leverages prior tactile knowledge to improve the GPC observation models of new objects.

### 4.2. Gaussian Process Classification

The Gaussian Process Classification (GPC) model describes the mapping between the observation set *X* and the output set *Y* by: $X\overset{f}{\to }Y$. The latent function g(x) in the GPC model is assumed to be sampled from a high-dimensional gaussian distribution called GP prior [46]: $g\left(x\right)∼\mathrm{GP}(m\left(x\right),K\left(x,x^{\prime }\right))$, where each sample g(x) is a random variable. In this work, we use one-vs-all multi-class classification. For each object class, a binary GPC whose output label is converted to {−1,+1} is trained for each of the *N* labels: $f_{n}\left(\cdot \right)$. Given a new sample $x^{*}$, each binary classifier predicts the observation probability of its label $p(y_{n}|x^{*})$. The sample is assigned to the class with the largest prediction probability $y^{*}=\arg\max_{y_{n}\in Y}\;p\left(y_{n}|x^{*}\right)$.

### 4.3. Process

**Figure 4:**
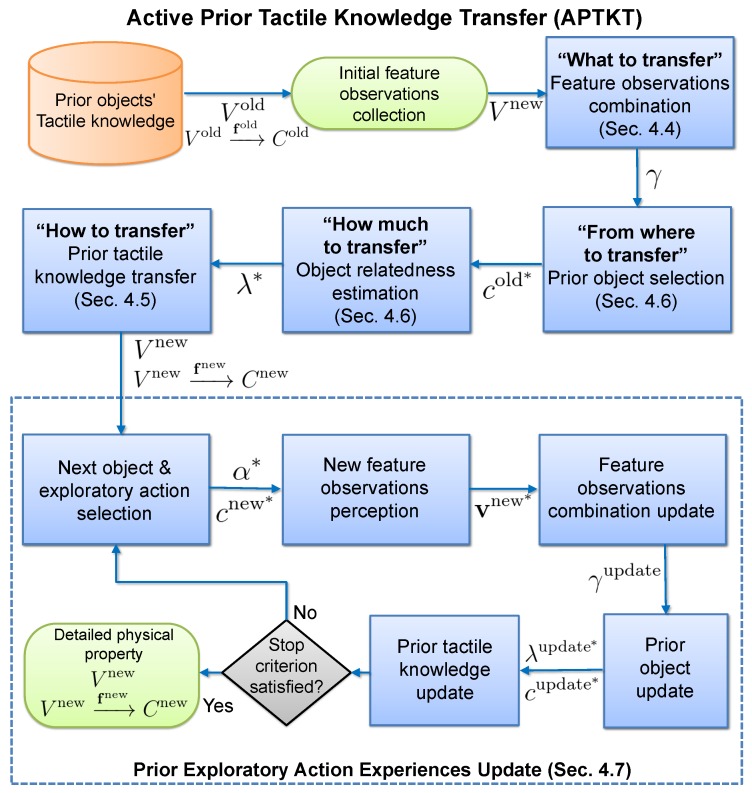
Flowchart of the Active Prior Tactile Knowledge Transfer algorithm.

The robot following our proposed method first applies each exploratory action one time on each new object, in order to collect a small number of feature observations $V^{\mathrm{new}}={\left\{V_{\alpha_{n}}^{\mathrm{new}}\right\}}_{n=1}^{N_{\alpha }}$ (Initial data collection). Then, the robot reuses its prior tactile exploratory action experiences to improve the observation models for *each* new object (Initial prior knowledge transfer). During this process, the robot compares the relatedness between its prior tactile exploratory action experiences and the new objects (Section 4.6), and chooses the most related one to transfer the old object feature observations $V^{\mathrm{old}}$ (Section 4.5). Afterwards, the robot begins to iteratively collect and combine the feature observations and update the prior tactile knowledge in order to improve the observation models. At each iteration of prior tactile knowledge updating, the robot (1) actively selects the next object and the next exploratory action in order to attain a new feature observation; and (2) updates the prior tactile knowledge for the selected exploratory action. The iteration terminates when there is no improvement in the observation models of new objects. Our algorithm is demonstrated by Figure 4.

### 4.4. What to Transfer

When the robotic system applies an exploratory action on objects, it perceives multiple feature observations (e.g., by the pressing movement, the robot can perceive the object stiffness and thermal conductivity). The prior tactile exploratory action experiences are built using the feature observations of prior objects from multiple sensory modalities that are combined together and the corresponding GPC observation models of prior objects.

**Figure 5:**
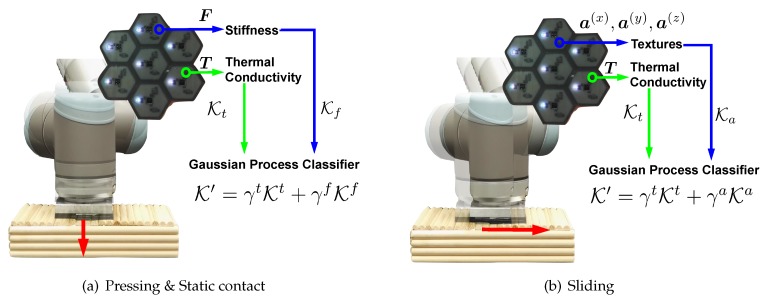
Illustration of multiple feature observations combination method. (**a**) The robotic system combines the normal force sensing and temperature sensing to learn about objects by applying pressing and static contact movements; (**b**) The robot slides on the object surface to sense its textural property and thermal conductivity.

In order to combine the observations perceived from different tactile sensors, we first define $v_{\alpha }$ as the feature observation of an exploratory action α. It is comprised of multiple observations: $v_{\alpha }=\left[v_{\alpha }^{\left(1\right)},\ldots ,v_{\alpha }^{\left(m_{\alpha }\right)},\ldots ,v_{\alpha }^{\left(M_{\alpha }\right)}\right]$, where $v_{\alpha }^{\left(m_{\alpha }\right)}$ is an observation from the sensor modality $m_{\alpha }$, $M_{\alpha }$ is the number of sensing modalities. For the pressing and static contact movements, we use the normal force and temperature sensing, for the sliding movement the accelerometer and temperature sensing (Table 2). Then, we assume that for a sensor modality $m_{\alpha }$, a kernel function $K^{\left(m_{\alpha }\right)}$ is given. To combine multiple feature observations so as to exploit the information from all sensors after applying the exploratory action α, we linearly combine the kernels:

(1) $$K_{\alpha }^{\prime }=\gamma_{\alpha }^{\left(1\right)}K^{\left(1\right)}+\ldots +\gamma_{\alpha }^{\left(m_{\alpha }\right)}K^{\left(m_{\alpha }\right)}+\ldots +\gamma_{\alpha }^{\left(M_{\alpha }\right)}K^{\left(M_{\alpha }\right)},$$

where $\gamma_{\alpha }^{\left(m_{\alpha }\right)}\geq 0$. This hyper-parameter controls how much the robot can rely on the sensor modality $m_{\alpha }$. It ranges between 0 and 1, with $\gamma_{\alpha }^{\left(m_{\alpha }\right)}=0$ indicating that the sensor feedback is not informative, and $\gamma_{\alpha }^{\left(m_{\alpha }\right)}=1$ highly useful. We further constrain these hyper-parameters with $L_{1}$ norm: $|\sum_{m_{\alpha =1}}^{M_{\alpha }}\gamma_{\alpha }^{\left(m_{\alpha }\right)}|=1.$ For each exploratory action, a GPC observation model is built using $K^{\prime }$. The hyper-parameters of γ and kernels are selected by maximizing the log marginal likelihood [46]. Figure 5 illustrates our multiple feature observations combination method. It is also demonstrated by Algorithm 1.

**Algorithm 1** Multiple Feature Observations Combination
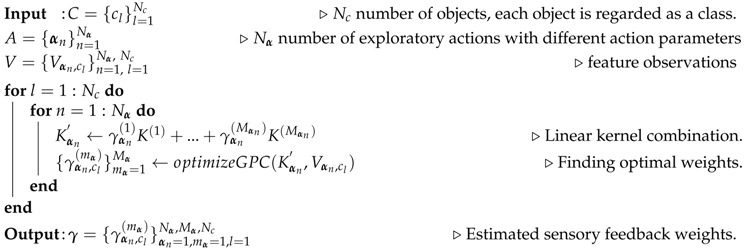


### 4.5. How to Transfer

Taking advantage of our previously proposed method [45], the robotic system transfers the feature observations of a prior object $c_{i}^{\mathrm{old}}$ to learn the GPC observation model of a new object $c_{j}^{\mathrm{new}}$, based on an exploratory action α. For simplicity, we hereby refer to *i* and *j* as $c_{i}^{\mathrm{old}}$ and $c_{j}^{\mathrm{new}}$, respectively. We define $g_{i}^{\mathrm{old}}$ as the Gaussian Process latent function values [46] for the old object $c_{i}^{\mathrm{old}}$ and $g_{j}^{\mathrm{new}}$ for the new object $c_{j}^{\mathrm{new}}$. We assume that these two function values are not independent of each other, but are sampled together over a dependent Gaussian Prior (GP). This dependent GP is then used to construct the GPC observation model of the new object. The latent function can be modified accordingly: $g_{j}^{{\mathrm{new}}^{\prime }}\leftarrow \left[g_{i}^{\mathrm{old}},g_{j}^{\mathrm{new}}\right]$ [45]. We further incorporate the relatedness between prior object and new object into the dependent GP model by introducing the following dependent kernel function:

(2) $$\begin{array}{c} K^{\prime }=\left[\begin{array}{cc} K(V_{i}^{\mathrm{old}},V_{i}^{\mathrm{old}})\;\;\;\;\; & \lambda K(V_{i}^{\mathrm{old}},V_{j}^{\mathrm{new}})\\ \lambda K(V_{j}^{\mathrm{new}},V_{i}^{\mathrm{old}})\;\;\;\;\; & K(V_{j}^{\mathrm{new}},V_{j}^{\mathrm{new}}) \end{array}\right]. \end{array}$$

$K(V_{i}^{\mathrm{old}},V_{i}^{\mathrm{old}})$ and $K(V_{j}^{\mathrm{new}},V_{j}^{\mathrm{new}})$ serve as the kernel matrix that measures the similarity among all feature observations of the old object and the new object, respectively. Each element in the kernel matrix measures the similarity between two feature observations, which is calculated by the radial basis function (RBF). $\lambda K(V_{j}^{\mathrm{new}},V_{i}^{\mathrm{old}})$ and $\lambda K(V_{i}^{\mathrm{old}},V_{j}^{\mathrm{new}})$ are the kernel matrix between the old object and the new object. λ controls the relatedness, or similarity, between $c_{i}^{\mathrm{old}}$ and $c_{j}^{\mathrm{new}}$. We constrain its range within [0,1]. As Chai et al. [47] evaluated, λ=0 indicates that the old object and the new object are totally different, while λ=1 indicates that the two objects are the same.

### 4.6. From Where and How Much to Transfer

Section 4.5 describes how to transfer the prior tactile knowledge to learn about new objects. This section illustrates how the robotic system selects the most related old object (from where to transfer) and how to determine the relatedness (λ) between two objects (how much to transfer).

To do this, we use our previously proposed method [45]. Let $p(c_{i}^{\mathrm{old}}|v_{j}^{\mathrm{new}})$ be the prediction probability that a feature observation from the new object $v_{j}^{\mathrm{new}}$ is assigned to the old object $c_{i}^{\mathrm{old}}$. We measure the average prediction to all the observations $v_{j}^{\mathrm{new}}\in V_{j}^{\mathrm{new}}$ that belong to the new object: $\bar{p}\left(c_{i}^{\mathrm{old}}|V_{j}^{\mathrm{new}}\right)=\frac{1}{N_{j}^{\mathrm{new}}}\sum p\left(c_{i}^{\mathrm{old}}|v_{j}^{\mathrm{new}}\right)$, with $N_{j}^{\mathrm{new}}$ being the number of new object feature observations. This average prediction value indicates the similarity between the old object $c_{i}^{\mathrm{old}}$ and the new object $c_{j}^{\mathrm{new}}$. A larger value indicates that these two objects are highly similar. Therefore, we can use it to select the most related old object (denoted as $c^{{\mathrm{old}}^{*}}$) for a new object regarding the exploratory action α. Furthermore, to avoid transferring irrelevant tactile information, we add a threshold $\epsilon_{neg}$ which prevents the robot from selecting any old object when the prediction value is smaller than $\epsilon_{neg}$. The final old object selection criterion is:

(3) $$\begin{array}{c} c^{{\mathrm{old}}^{*}}=\left\{\begin{array}{cc} \underset{c_{i}^{\mathrm{old}}\in C^{\mathrm{old}}}{\arg\max}\;\bar{p}\left(c_{i}^{\mathrm{old}}|V_{j}^{\mathrm{new}}\right), & \mathrm{if}\;\;\;\bar{p}\left(c^{{\mathrm{old}}^{*}}|V_{j}^{\mathrm{new}}\right)\geq \epsilon_{neg}\\ \mathrm{None}, & \mathrm{otherwise}. \end{array}\right. \end{array}$$

Once we select $c^{{\mathrm{old}}^{*}}$, we further use the predictions from the observation model of old objects to determine the object relatedness $\lambda^{*}$: $\lambda^{*}=\bar{p}\left(c^{{\mathrm{old}}^{*}}|V^{\mathrm{new}}\right)$.

### 4.7. Prior Exploratory Action Experiences Update

When the robot updates its prior exploratory action experiences, it needs to iteratively collect a new feature observation by applying an exploratory action on an object. We use our previously proposed active tactile learning algorithm [43] called Active Touch for Learning Physical Properties (AT-LPP). Using our AT-LPP approach, the robot actively decides which new feature on the object to explore next (denoted as $c^{{\mathrm{new}}^{*}}$) and which physical property to learn next (which exploratory action to apply next). It is denoted as $\alpha^{*})$. In the following, we briefly summarize the AT-LPP algorithm (Algorithm 2) [43].

The robot first calculates the Shannon entropy of the object posterior for a new feature observation $v^{\mathrm{new}}$ with the equation: $H\left(c^{\mathrm{new}}|v^{\mathrm{new}}\right)=-\sum_{c_{j}^{\mathrm{new}}\in C^{\mathrm{new}}}p\left(c_{j}^{\mathrm{new}}|v^{\mathrm{new}}\right)\log\left(p\left(c_{j}^{\mathrm{new}}|v^{\mathrm{new}}\right)\right)$. Then the robot estimates the uncertainty in the GPC model with regard to each exploratory action and new object by the mean value of the Shannon entropy: $\mathrm{UNC}\left(\alpha_{n},c_{j}\right)=\frac{1}{N_{\alpha_{n},j}^{\mathrm{new}}}\sum_{v_{\alpha_{n},j}^{\mathrm{new}}\in V_{\alpha_{n},c_{j}^{\mathrm{new}}}^{\mathrm{new}}}H\left(c_{j}^{\mathrm{new}}|v_{\alpha_{n},j}^{\mathrm{new}}\right)$, where $v_{\alpha_{n},j}^{\mathrm{new}}$ refers to the a feature observation the robot has collected for the new object $c_{j}^{\mathrm{new}}$ and exploratory action $\alpha_{n}$; $N_{\alpha_{n},j}^{\mathrm{new}}$ is the number of feature observations. A large $\mathrm{UNC}(\alpha_{n},c_{j})$ indicates that the robot is uncertain about the object feature observations from the exploratory action $\alpha_{n}$. As discussed in [43], an efficient next object and the next action selection process should be considered to greedily reduce such uncertainty while at the same time allowing the robot to explore (exploration-exploitation trade-off). In this regard, the next exploratory action $\alpha^{*}$ and the next object $c^{{\mathrm{new}}^{*}}$ are determined by:

(4) $$\begin{array}{c} \left\{\begin{array}{cc} c^{{\mathrm{new}}^{*}},\alpha^{*}=\underset{\alpha_{n}\in A;\;c_{j}^{\mathrm{new}}\in C^{\mathrm{new}}}{\arg\max}\mathrm{UNC}\left(\alpha_{n},c_{j}^{\mathrm{new}}\right)\;\;\; & \mathrm{if}\;p_{\mathrm{rand}}\geq \epsilon_{\mathrm{explor}}\\ c^{{\mathrm{new}}^{*}}=U\left\{c_{1}^{\mathrm{new}},c_{2}^{\mathrm{new}},\ldots ,c_{N_{\mathrm{new}}}^{\mathrm{new}}\right\},\alpha^{*}=U\left\{\alpha_{1},\alpha_{2},\ldots \alpha_{N_{\alpha }}\right\} & \mathrm{otherwise}, \end{array}\right. \end{array}$$

where $\epsilon_{\mathrm{explor}}$ is the exploration rate, and $p_{\mathrm{rand}}$ is randomly generated following the uniform distribution U(0,1).

**Algorithm 2** Active Touch for Learning Physical Properties
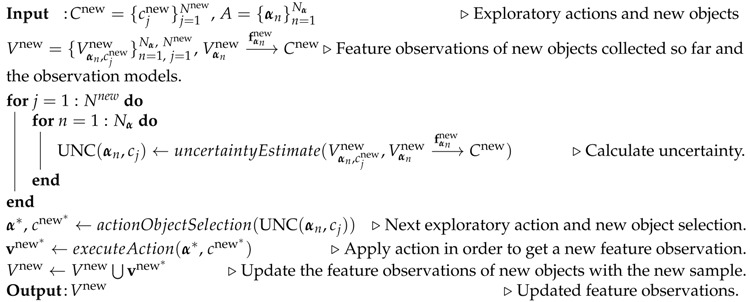


Once the robot collects a new feature observation, it updates the prior tactile exploratory action experiences only from action $\alpha^{*}$. This process includes updating the feature observation combination, updating the object relatedness λ, and transferring these prior feature observations to the observation models of new objects.

## 5. Experimental Results

### 5.1. Experimental Objects

In order to evaluate the performance of the proposed active prior tactile knowledge transfer algorithm (APTKT), we deliberately selected 10 daily objects with different physical properties which served to build the robotic prior to tactile exploratory action experiences (see Figure 1 Prior objects). Furthermore, we selected five new objects about which the robotic system was tasked to learn (Figure 1 New objects). For each new object, there existed one or more old objects that shared similar physical properties. For example, both rough sponge and smooth sponge are soft; paper box and hard box have similar surface textures; metal toolbox and biscuit box have high thermal conductivity. In this way, when learning about new objects based on their physical properties, the robot can leverage the related prior tactile knowledge.

### 5.2. Exploratory Action Determination and Test Data Collection

In our experiment, we defined seven exploratory actions from the pressing, sliding, and static contact movements with various action parameters (Pressing: P1, $d_{P}=1$ mm, $t_{P}=3$ s; P2, $d_{P}=2$ mm, $t_{P}=3$ s. Sliding: S1, $F_{S}=0.1$ N, $t_{S}=5$ s $v_{S}=1$ cm/s; S2, $F_{S}=0.1$ N, $t_{S}=1$ s, $v_{S}=5$ cm/s; S3, $F_{S}=0.2$ N, $t_{S}=5$ s, $v_{S}=1$ cm/s; S4, $F_{S}=0.2$ N, $t_{S}=1$ s, $v_{S}=5$ cm/s. Static Contact: C1, $d_{C}=2$ mm, $t_{C}=15$ s). Before applying any of the seven exploratory actions, the robot established light contact with the objects which were detected once the total normal force on the artificial skin increased above 0.05 N. Furthermore, after applying an exploratory action, the robot was controlled to raise its end-effector for 30 s such that the temperature sensors could be restored to the ambient temperature.

We evaluated the performance of our proposed method based on a test dataset built by the robot by applying each actions 20 times on each object. During this process, objects were manually shifted and rotated so that the data was robust against the variations in the object contact locations with the artificial skin.

### 5.3. Evaluation of Multiple Feature Observations Combination Method

We first evaluated the performance of our proposed robotic multiple feature observation combination algorithm. To do this, the robot selected 10 groups of objects (shown in Figure 1) to construct the GPC observation models for each of the seven exploratory actions. Each group contained five objects that were selected randomly both from the old and new object lists, following a uniform distribution. The algorithm performance was evaluated by the discrimination accuracy of the test dataset predicted by the GPC models with the growing number of feature observations. We compared our method with the baseline methods that built the GPC models using only a single sensor modality.

**Figure 6:**
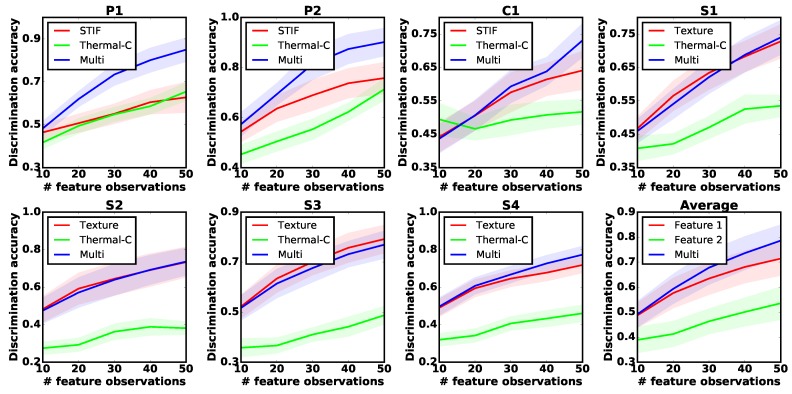
Multiple feature observations combination results for exploratory actions P1, P2, C1, S1, S2, S3, S4 and the averaged result. STIF: building the GPC observation model based on object stiffness; Thermal-C: thermal conductivity; Texture: object surface textural properties; Multi: combined feature observations. The horizontal axis represents the number of feature observations. The vertical axis represents the discrimination accuracy of the test dataset.

The experiments were conducted 10 times for each object group. For a fair comparison, we used RBF kernel [46] for each sensor modality. Results are plotted in Figure 6. For all seven exploratory actions, our proposed algorithm either took advantage of combining different sensor modalities to reach the best discrimination accuracy (P1, P2, C1, S4 in Figure 6), or performed the same as the best single-sensor result (S1, S2, S3 in Figure 6), indicating that the robot actively selected the most informative sensory feedback to learn about objects.

### 5.4. Evaluation of the Transfer Learning Method with Different Groups of Prior Objects

In this experiment, we evaluated the performance of our proposed transfer learning method (APTKT) to learn the five new objects (see new objects in Figure 1) with different groups of prior objects (see prior objects in Figure 1). To start the learning process, the robot applied each of the seven actions once on each new object. When the robot iteratively learned the new objects’ physical properties, it updated both the multiple feature observations combination and the prior tactile knowledge built by the dependent GPC models with all the feature observations collected so far. At each learning iteration, we measured the object discrimination accuracy of the test dataset. The transfer learning performance was compared with the baseline learning method that combined multiple feature observations without transferring any prior tactile knowledge.

**Figure 7:**
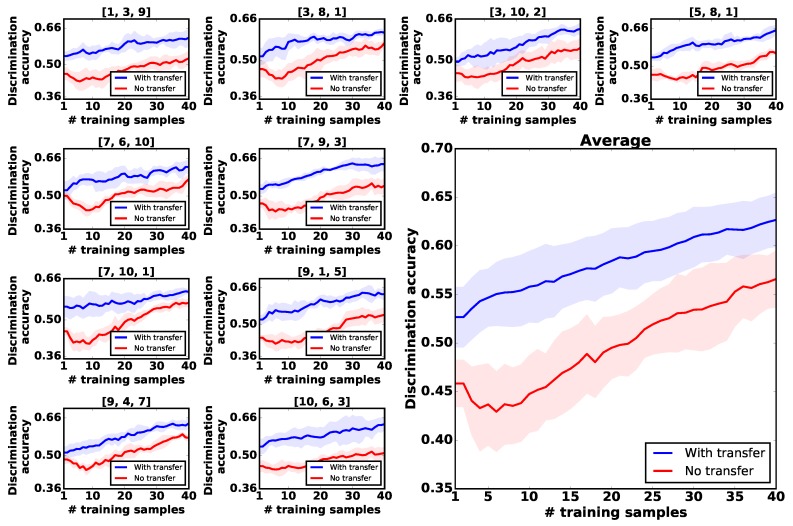
Transferring the exploratory actions experiences from three prior objects. The small plots show the learning process from 10 groups of old objects. The large plot on the right shows the averaged results. Horizontal axis: the growing number of feature observations the robot collected. Vertical axis: the discrimination accuracy of the test dataset.

We randomly shuffled the prior objects into ten groups following a uniform distribution. Each group consisted of the feature observations and the observation models from three prior objects. We conducted the experiment with five trials for each group. In each trial, the robot followed the transfer learning approach and no-transfer approach to collect 40 feature observations in total, allowing a fair comparison between different learning strategies to be made. Figure 7 illustrates that with the help of prior knowledge, the robot consistently outperformed the learning process without prior knowledge with a discrimination accuracy of 10%.

**Figure 8:**
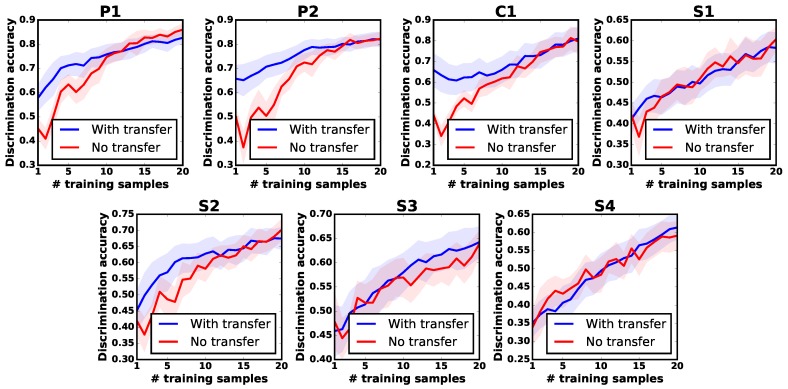
Transfer learning using only one exploratory action.

In order to further evaluate the robustness of APTKT, the robot was then tasked to learn about objects via applying only *one* of the exploratory actions. The experimental procedure was the same as the one described above. As the results in Figure 8 show, The robot had a larger improvement by actions P1, P2 and C1 than actions S1, S2, S3 and S4. For example, the robot increased the discrimination accuracy by 25%, when it reused the prior tactile instance knowledge from the movement P2. However, when learning about objects by actions S1 and S4, little improvement was seen. This was due to the fact that different exploratory actions produced different object feature observations. For action P2, there existed higher related prior tactile knowledge than S1 and S4, and the robot could benefit more on it.

In all scenarios, using our proposed transfer learning algorithm, the robot could achieve a higher discrimination accuracy than the baseline method with the same number of feature observations. Therefore, we can conclude that APTKT helps the robot build reliable observation models of new objects with fewer training samples, even when only one kind of exploratory action is applied.

### 5.5. Increasing the Number of Prior Objects

**Figure 9:**
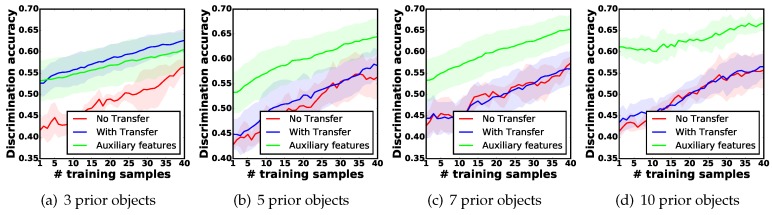
Increasing the number of prior objects from 3, 5, 7 to 10, and comparing the performance of different learning methods. Red: baseline method; Blue: the proposed active prior tactile knowledge transfer method (APTKT) without auxiliary features; Green: APTKT with auxiliary features.

We further evaluated the performance of our proposed method with an increasing number of prior tactile experiences. Intuitively, as the number of old objects grows, it is more likely that the robot can find highly-related prior tactile knowledge, so that the learning performance can continue to be improved. In this regard, the robot was asked to learn about new objects via all seven exploratory actions, with the number of old objects increasing from 5, 7 to 10. We followed the same experimental procedure described above, and conducted each experiment with five trials. Unexpectedly, as Figure 9b–d show, the growing number of prior tactile knowledge reduced the transfer learning improvement. This was because as the number of prior objects grow, it was more difficult for the robot to classify them. As a result, the object relatedness λ predicted by the old object GPC models was lower than the threshold $\epsilon_{\mathrm{neg}}$, making the robot stop transferring prior knowledge.

To compensate for this, we use our previously proposed feature augmentation trick [45]. We defined $p(c_{i}^{\mathrm{old}}|v)$ as the prediction probability that a feature observation from the new object v is assigned to the old object $c_{i}^{\mathrm{old}}$. Then we augmented a feature observation v from a new object as:

(5) $$\begin{array}{c} v^{\prime }=\left[\underset{\mathrm{original}\;\mathrm{features}}{\underset{︸}{v,}}\;\;\;\;\underset{\mathrm{predictions}\;\mathrm{from}\;\mathrm{old}\;\mathrm{objects}{}^{\prime }\;\mathrm{observation}\;\mathrm{models}}{\underset{︸}{p\left(c_{1}^{\mathrm{old}}|v\right),\ldots ,p\left(c_{i}^{\mathrm{old}}|v\right),\ldots ,p\left(c_{N_{\mathrm{old}}}^{\mathrm{old}}|x\right)}}\right]. \end{array}$$

The auxiliary features $\left[p\left(c_{1}^{\mathrm{old}}|v\right),\ldots ,p\left(c_{N_{\mathrm{old}}}^{\mathrm{old}}|v\right)\right]$ encode the knowledge of all prior objects. They represent the relatedness between prior objects and the new object, and thus can help the robotic system to distinguish among new objects. Furthermore, since the auxiliary features can be regarded to be perceived from an auxiliary sensor, we directly employed our proposed multiple feature observation combination method to the augmented feature observations by casting a weight γ to its kernel. The augmented feature observations were then used to build the new object dependent GPC models.

We tested our proposed feature augmentation technique when the robot leveraged the tactile knowledge of 3, 5, 7, and 10 prior objects to learn about new objects via all seven actions. The learning performance is shown by the green curves in Figure 9a–d. Clearly, by introducing the probability predictions as auxiliary features, the robot was able to reuse the prior tactile knowledge again, and it achieved similar improvement of discrimination accuracy for 3 prior objects, and higher improvement for 5, 7, and 10 prior objects compared to the other methods. Specifically, when resuing 10 prior objects, the robot achieved 20% higher discrimination accuracy than the baseline method, when only *one* new feature observation was collected, showing the one-shot learning behaviour. This experiment also indicates that with a further growing number of prior objects, a further improvement of discrimination accuracy is achievable.

### 5.6. Negative Prior Tactile Knowledge Transfer Testing

When the constructed prior tactile exploratory action experiences are not relevant to the new objects, a brutal-force transfer may degrade the learning performance, resulting in the negative knowledge transfer phenomena. In this case, the transfer learning algorithm should stop leveraging irrelevant prior knowledge.

**Figure 10:**
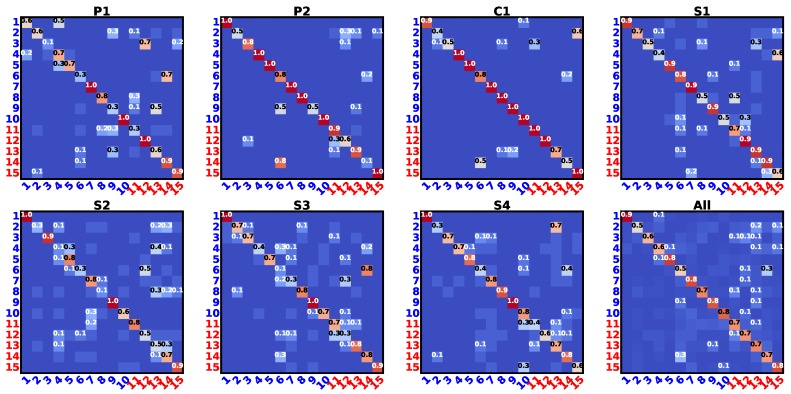
object confusion matrices (value normalized between 0 and 1) for each exploratory action and the average. The blue indices represent the old objects. The red indices represent the new objects, with #11–#15 indicating new objects #1–#5. Best viewed in magnification.

In order to evaluate our proposed transfer learning method (APTKT) against the negative tactile knowledge transfer, we deliberately selected irrelevant prior objects and compared the transfer learning performance with the baseline method, following the same experimental process described in Section 5.4. When finding which objects were relevant (or irrelevant) to each other, we built object confusion matrices to roughly evaluate the object similarity. For each of the seven exploratory actions, we trained a Gaussian Mixture Model (GMM) and calculated the object confusion matrix. To do this, we first used GMM to cluster all the samples from the dataset with the hyper-parameters optimized by the Expectation-Maximization (EM) algorithm. The number of clusters was set to be the same as the number of objects (in our case, 15), and each cluster centroid was initialized as the mean value of all data samples that belonged to an object. The maximum EM iterations was set to be 100, with convergence threshold being 0.001. We further calculated the confusion matrix averaged over all exploratory actions. These matrices indicated the averaged similarity between objects. We rescaled their values to be within 0-1, with 0 meaning that two objects are totally dissimilar, and 1 the same. The objects which had low similarity values with target objects were selected as irrelevant objects. The results are shown in Figure 10. According to Figure 10, prior objects {1, 5, 7} (objects {1–10}) were dissimilar to the new objects (objects {11–15}) regarding the exploratory movement P1, objects {1, 4, 7} for P2, objects {4, 7, 10} for C1, objects {1, 6, 9} for S1, objects {1, 7, 10} for S2, objects {1, 3, 9} for S3, and objects {1, 3, 8} for S4. We thus used these objects as prior objects to test the transfer learning performance via the single exploratory action. We further selected objects {1, 5, 10} to test the learning process via all exploratory actions, since these three objects shared relative small similarity to the new objects.

**Figure 11:**
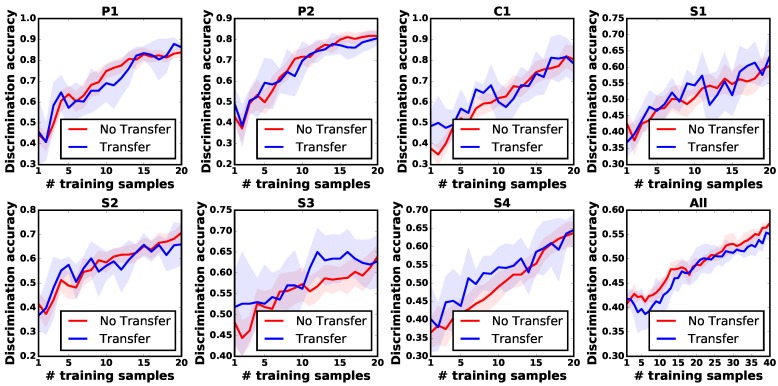
Negative prior tactile knowledge transfer testing. The prior objects that were unrelated to the new objects were deliberately selected.

The results in Figure 11 illustrate that the discrimination accuracy achieved by APTKT was similar to the baseline method, when the robot applied either one or all seven exploratory actions. The results indicate that our proposed algorithm stopped transferring negative prior tactile instance knowledge.

## 6. Conclusions

In this work, we proposed a transfer learning method for a robot equipped with multi-modal artificial skin to actively reuse the prior tactile exploratory action experiences when learning about the detailed physical properties of new objects. These prior action experiences are built by the feature observations, when the robotic arm applies the pressing, sliding and static contact movements with different action parameters on the previous-explored objects (prior objects). The feature observations are perceived from multiple sensory modalities. Using our proposed tactile transfer learning method, the robot has a "warm start" of the learning process. It applies fewer exploratory actions and gains a detailed tactile knowledge of new objects (e.g., normal force feedback at different pressing depths).

One limitation of our work is that performing static contact movement took 15 *s*, which prevented the rapid transfer learning. Furthermore, due to the limitations of our artificial skin, the robot can only interact with objects with flat surfaces. In the future, we will extend our method to more exploratory actions (such as tapping and lifting), so that the robot can transfer more exploratory action experiences to learn more physical properties of an object, such as auditory feedback and center of mass. Furthermore, an interesting topic would be how to transfer the prior tactile knowledge across different exploratory actions, e.g., transferring the tactile knowledge from pressing to static contact movement.

## Figures and Tables

**Table 1:** Technical information of sensors in the artificial skin ([12]).

Type	Sensor	Range	Accuracy	Resolution
Proximity	VCNL4010	200mm	N.A.	0.25 lx
Acceleration	BMA250	±2g	256LSB/g	3.91 mg
Temperature	LM71	−40–150 °C	$\pm 1.5{\;}^{\circ }C$	$31.25\;m^{\circ }C$
Normal force	customized	>10N	0.05N	N.A.

**Table 2:** Exploratory actions and perception.

Exploratory actions	Action Parameters (θ)	Sensory feedbacks	Features
Pressing	$d_{P}$, $t_{P}$	F, T	$\bar{F}$, $\left[\bar{T},\nabla \bar{T}\right]$
Sliding	$F_{S}$, $t_{S}$, $v_{S}$	a, T	TD, $\left[\bar{T},\nabla \bar{T}\right]$
Static contact	$d_{C}$, $t_{C}$	F, T	$\bar{F}$, $\left[\bar{T},\nabla \bar{T}\right]$
